# Combination of Au-Ag Plasmonic Nanoparticles of Varied Compositions with Carbon Nitride for Enhanced Photocatalytic Degradation of Ibuprofen under Visible Light

**DOI:** 10.3390/ma14143912

**Published:** 2021-07-14

**Authors:** Marta Jiménez-Salcedo, Miguel Monge, María Teresa Tena

**Affiliations:** Centro de Investigación en Síntesis Química (CISQ), Department of Chemistry, University of La Rioja, Complejo Científico-Tecnológico, 26006 Logrono, Spain; marta.jimenez@unirioja.es (M.J.-S.); maria-teresa.tena@unirioja.es (M.T.T.)

**Keywords:** gold, silver, carbon nitride, nanostructures, ibuprofen, photocatalysis, visible light

## Abstract

Au-Ag/g-C_3_N_4_ nanohybrids **2**–**3** were synthesized by the one-pot self-reduction of the organometallic precursor [Au_2_Ag_2_(C_6_F_5_)_4_(OEt_2_)_2_]_n_ in the presence of graphitic carbon nitride (g-C_3_N_4_), leading to two populations of alloyed Au-Ag nanoparticles (NPs) of different size and composition on the surface of g-C_3_N_4_, i.e., Ag-enriched Au-Ag NPs of smaller size and Au-enriched Au-Ag NPs of larger size. The combination of these two types of plasmonic NPs with g-C_3_N_4_ semiconductor displays enhanced photocatalytic properties towards the degradation of ibuprofen under visible light by the increased charge carrier separation provided by the inclusion of the plasmonic NPs on g-C_3_N_4_.

## 1. Introduction

During the last years the search for efficient photocatalytic materials designed for sustainable applications such as photovoltaic devices, water splitting, photocatalytic chemical transformations or degradation of pollutants have attracted wide attention [1,2]. Extensive research on the development and applications of titania-based semiconductor photocatalysts under ultraviolet light has been carried out since the 1970s [3], but their lack of or reduced use of visible light, which is the main component of solar radiation on Earth, has prompted the research community to focus on the development of new visible light-active materials as efficient photocatalysts [4,5,6]. At this regard, taking into account that the solar light is formed by ultraviolet (UV) (5%), visible (43%) and infrared (IR) (52%) components, many of the known photocatalysts present low overall solar energy utilization [7], making the design of novel photocatalysts displaying high efficiencies under visible or near infrared (NIR) light excitation and their use in the degradation of water pollutants a very challenging issue [8].

Among the wide family of semiconductors used in photocatalysis for the degradation of pollutants the narrow band-gap semiconductor graphitic carbon nitride (g-C_3_N_4_) has emerged as an outstanding material due to a number of interesting features [9], namely: (i) visible light response (band-gap of ca. 2.7 eV); (ii) a sheet-like nanostructure displaying a high surface to volume ratio; (iii) thermal and chemical stability; (iv) good adsorption ability and (v) facile synthesis. However, this material also displays important drawbacks such as a narrow visible light absorption window or the fast recombination of charge carriers.

A smart approach to overcome these disadvantages consists of the formation or deposition of noble gold (Au) or silver (Ag) or even to a lesser extent bimetallic Au-Ag metal nanoparticles (NPs) on the surface of g-C_3_N_4_ nanosheets [10,11]. These hybrid nanosystems constitute a unique challenge in which the catalytic, electronic and optical properties of the metal nanoparticles are combined with the abovementioned ones provided by the g-C_3_N_4_ nanosheets. Indeed, on the one hand these metals display strong size and shape-dependent light absorption in the visible or NIR ranges due to the localized surface plasmon resonance effect (LSPR) [12,13,14,15], i.e., the resonant oscillation of conduction electrons in response to an oscillating electric field from an incident electromagnetic radiation [16,17,18]. This effect has given rise to the study of interesting related properties such as plasmonic photocatalysis, plasmon-enhanced fluorescence, surface enhanced Raman scattering (SERS) or photothermal heating [19]. The combination of these plasmonic NPs with g-C_3_N_4_ allows one to synergistically overcome some of the main drawbacks of the pristine semiconductor. Thus, LSPR effects such as local heating, near-field effect or charge-transfer processes of plasmonically excited electrons (hot electrons) to the semiconductor cut out to a large extent the electron-hole recombination [20]. On the other hand, and very importantly, when noble metal NPs are grafted at the semiconductor surface a contact potential is formed in the heterojunction, which is called the Schottky barrier. This energy barrier enables a unidirectional transfer of photogenerated electrons in the semiconductor to the metal NPs, which act as electron sinks, leading to a very effective charge carrier separation [21]. This enhanced charge carrier separation greatly favors the formation of reactive oxygen species (ROS), leading to an improved photocatalytic performance.

There are several approaches for the synthesis of metal NPs-g-C_3_N_4_ nanohybrids, which include pulsed laser ablation, hydro/solvothermal techniques, microwave assisted synthesis, chemical reduction, wet impregnation/calcination or photodeposition, being the latter the most convenient approach [11].

We have focused a part of our research efforts on the synthesis of bimetallic Au-Ag plasmonic nanostructures through an organometallic approach. This method consists of the self-reduction under mild reaction conditions of pentafluorophenyl-based precursors through a bimolecular reductive elimination mechanism, which allows one to exert an excellent control over the size, shape, composition and surface state of the NPs. In order, to exert this control, different types of capping agents such as long-alkyl chain ligands [22] or polymers can be used [23]. However, the use of these growth directing agents usually prevents the utilization of such nanostructures as (photo)catalysts, although they present interesting tunable plasmonic properties. In addition, a previous study by some of us on the UV-vis time-monitored formation of alloy Au-Ag NPs by decomposition of complex [Au_2_Ag_2_(C_6_F_5_)_4_(OEt_2_)_2_]_n_ in the presence of hexadecylamine as capping ligand [22], showed that this decomposition was sequential, starting from less stable Ag(I) intermediates and followed by Au(I) ones, formed in situ. This approach allowed the synthesis of Au-Ag NPs with distinct metal contents, leading to a tuning of the plasmonic properties. 

At this point we wondered whether this controlled sequential decomposition of the organometallic precursor [Au_2_Ag_2_(C_6_F_5_)_4_(OEt_2_)_2_]_n_ would give rise to the formation of composition-controlled Au-Ag NPs stabilized at the surface of g-C_3_N_4_, without the concurrence of any other growth directing ligand or polymer, leading to small-size plasmonic nanoparticles of different gold/silver content efficiently grafted to the surface of g-C_3_N_4_. This approach would provide several advantages, namely: (i) the triazine groups of g-C_3_N_4_ would be able to stabilize Au-Ag NPs of different compositions formed during their sequential formation, (ii) a broader plasmonic absorption arising from the different Au-Ag NP compositions would be obtained, increasing the visible-light absorption ability and improving LSPR effects upon excitation with broad-range visible light and (iii) AuAg NPs of different compositions may act as electron sinks of photogenerated electrons improving the charge carrier and enhancing the photocatalytic performance towards the degradation of pharmaceuticals.

Herein we report the synthesis and in-depth characterization of plasmonic Au-Ag/g-C_3_N_4_ nanohybrids by the direct formation of alloy Au-Ag NPs of different compositions at the surface of 2D g-C_3_N_4_ nanosheets. We have also checked the photocatalytic performance of these NPs in the degradation of ibuprofen, including a study of the photocatalytic degradation mechanism.

## 2. Materials and Methods

### 2.1. Materials and Reagents

Ibuprofen, *tert*-butanol and triethanolamine were obtained from Sigma Aldrich (Schnelldorf, Germany). Melamine (99%) was provided by Alfa Aesar (Kandel, Germany). HPLC-grade acetonitrile was purchased from Scharlab (Barcelona, Spain) and formic acid (98% *v*/*v*) for mass spectrometry from Fluka Analytical (Buchs, Switzerland). Ethylene glycol and ethanol, used in the synthesis of nanoparticles, were provided from Sigma Aldrich and Panreac (Barcelona, Spain), respectively. All chemicals were used as received and no further purification was needed. The precursor [Au_2_Ag_2_(C_6_F_5_)_4_(OEt_2_)_2_]_n_ used for the synthesis of bimetallic AuAg NPs was synthesized by standard procedures reported in [24].

### 2.2. Synthesis of Photocatalysts

#### 2.2.1. Synthesis of g-C_3_N_4_ Nanosheets (**1**)

The preparation of the g-C_3_N_4_ powder was performed by the polycondensation of melamine. Melamine (5 g) was thermally treated in an oven at 500 °C for 4 h and then, the temperature was raised to 520 °C for 2 h in order to obtain exfoliated g-C_3_N_4_ nanosheets, as reported previously [25]. The reaction was carried out in a covered crucible in order to prevent partially the sublimation of the precursor.

#### 2.2.2. Synthesis of 0.5% Au-Ag-g-C_3_N_4_ Nanohybrid (**2**)

To a 30 min sonicated (40 kHz and 150 W) suspension of g-C_3_N_4_ (0.4253 g) in 5 mL of ethylene glycol, 0.5 wt% (metal content) [Au_2_Ag_2_(C_6_F_5_)_4_(OEt_2_)_2_]_n_ (5 mg, 3.51 × 10^−3^ mmol) was added and sonicated for 3 min. The suspension was stirred and heated under reflux at 130 °C, in dark conditions, during 15 min. After that, the formed nanohybrid was filtered and washed with ethanol. Finally, it was dried under reduced pressure, leading to a pale brownish solid.

#### 2.2.3. Synthesis of 1% Au-Ag-g-C_3_N_4_ Nanohybrid (**3**)

To a 30 min sonicated (40 kHz and 150 W) suspension of g-C_3_N_4_ (0.2116 g) in 5 mL of ethyleneglycol, 1 wt% (metal content) [Au_2_Ag_2_(C_6_F_5_)_4_(OEt_2_)_2_]_n_ (5 mg, 3.51 × 10^−3^ mmol) was added and sonicated for 3 min. The suspension was stirred and heated under reflux at 130 °C, in dark conditions, during 15 min. After that, the formed nanohybrid was filtered and washed with ethanol. Finally, it was dried under reduced pressure, leading to a pale brownish solid.

### 2.3. Characterization

The NMR spectra were recorded on a Avance 400 spectrometer (Bruker, Billerica, MA, USA) in THF-d_8_ solutions. Chemical shifts are expressed relative to CFCl_3_ (^19^F external reference). Diffuse reflectance UV−vis spectra of pressed powder samples diluted with silica were recorded on a UV-3600 spectrophotometer (Shimadzu, Kioto, Japan) equipped with a Harrick Praying Mantis accessory and recalculated following the Kubelka−Munk function. Samples for Transmission Electron Microscopy (TEM) were directly drop-casted from the ethanol dispersions (2–3 drops) to carbon-coated Cu grids. The TEM images were obtained with a JEM 2100 microscope (JEOL, Tokyo, Japan). High Angle Annular Dark Field—Scanning Transmission Electron Microscopy (HAADF-STEM) images were obtained in a Tecnai F30 (ThermoFisher Scientific, Waltham, MA, USA) or in a Cs-probe-corrected Titan (ThermoFisher Scientific, Waltham, MA, USA) instrument at a working voltage of 300 kV, coupled with a HAADF detector (Fischione, Export, PA, USA). In this mode, the intensity of the signal is proportional to the square of the atomic number (Z2), therefore heavier elements appear with a much brighter contrast than lighter elements, like carbon or silicon. It is especially useful to localize metals in organic matrixes. Also, in order to analyze the chemical composition of the materials, X-ray Energy Dispersive Spectra (EDS) were obtained with an EDAX detector or with an Ultim Max detector (Oxford, Oxford, Abingdon, UK). XPS experiments were performed on an AXIS Supra spectrometer (Kratos, Manchester, UK) using a monochromatized Al Ka source (1486.6 eV) operating at 12 kV and 10 mA. Wide scans were acquired at analyzer pass energy of 160 eV, whereas high-resolution narrow scans were performed at constant pass energy of 20 eV and steps of 0.1 eV. The photoelectrons were detected at a take-off angle of F = 0° with respect to the surface normal. Basal pressure in the analysis chamber was less than 5 × 10^–9^ Torr. The spectra were obtained at room temperature. The binding energy (BE) scale was internally referenced to the C 1s peak (BE for C − C =284.9 eV).

### 2.4. Photocatalytic Activity Measurement

In a typical ibuprofen photodegradation process, 134.5 mg of photocatalyst was suspended in 50 mL of a solution of ibuprofen in ultrapure water with a concentration of 5 µg mL^−1^ in a Schlenk tube glass reactor. Before starting the photocatalytic reactions, the suspensions were sonicated for 2 min, following by 40 min of stirring in dark conditions to reach adsorption/desorption equilibrium in the aqueous solution. 

Ibuprofen solutions were irradiated with visible light radiation in a cooled lab-made setup. The assembly consists of four 10 W white light LED lamps (LED-Engin, San Jose, CA, USA) arranged equidistantly inside a cylinder. Water recirculating coolant coil is placed on the outside to maintain constant temperature (25 °C) of the glass reactor.

In addition, we performed a series of experiments using natural sunlight in order to compare the performance of the catalysts 1–3 under these conditions using ibuprofen as wastewater pollutant. These experiments were carried out with an average temperature of 28 °C and an ultraviolet index of 7 accordingly with the Spanish National Agency of Meteorology (www.AEMET.es, accessed on 4 September 2020).

Photocatalytic reactions were performed stirring at natural pH 1.5 mL sample was taken regularly and filtered before analysis with nylon syringe filter 13 mm, 0.22 µm. Samples were stored at 4 °C until analysis by HPLC-UV.

### 2.5. Photodegradation Analysis

The ibuprofen concentration was analyzed using an Agilent modular 1100/1200 liquid chromatography system (Agilent Technologies, Palo Alto, CA, USA) equipped with a G1329A autosampler and a G1315D diode array detector and a Phenomenex Luna^®^ LC C18 100 Å (5 µm particle size, 150 mm × 4.6 mm i.d.) column. The mobile phase consisted of a 70:30 mixture of 0.1% formic acid in acetonitrile and 0.1% formic acid aqueous solution with a flow rate of 1.0 mL/min and an injection volume of 20 µL. Detection wavelengths were 220 nm.

### 2.6. Detection of Active Species in the Photocatalysis

To study the effect of active species responsible for the photodegradation using the catalyst doped with Au-Ag, the corresponding scavengers were introduced in a typical degradation to quench the specific reactive species: tert-butanol (1 mM) for OH radicals, triethanolamine (1 mM) to quench the holes (h^+^) and the reaction was performed under N_2_ atmosphere for ·O_2_^−^ quenching. These experiments were performed using the white light source from four 10 W white light LED lamps.

## 3. Results and Discussion

### 3.1. Synthesis and Characterization of Photocatalysts

In this work we have pursued the synthesis of AuAg-g-C_3_N_4_ nanohybrids **2–3** by the decomposition of the organometallic complex [Au_2_Ag_2_(C_6_F_5_)_4_(OEt_2_)_2_]_n_ (0.5 wt% of added metal content in the Au-Ag precursor (**2**) or 1 wt% (**3**)) over g-C_3_N_4_ sheets at 130 °C in ethyleneglycol as solvent and co-reducing agent. 

**Scheme 1:**
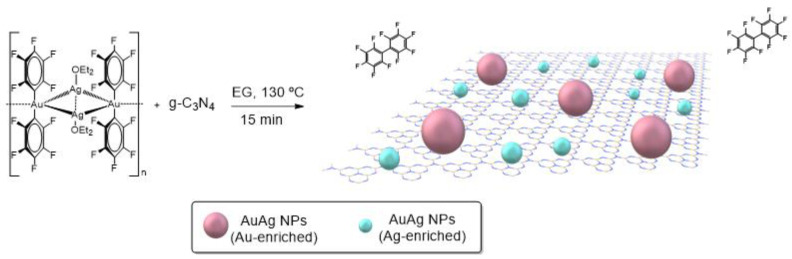
Synthesis of AuAg-g-C_3_N_4_ nanohybrids **2** and **3** by reduction of complex [Au_2_Ag_2_(C_6_F_5_)_4_(OEt_2_)_2_]_n_ in ethyleneglycol (EG) at 130 °C.

This novel approach provides the possibility of taking advantage of the presence of N-donor atoms in the triazine groups of the g-C_3_N_4_ nanosheets to effectively stabilize sub-10 nm size Au-Ag NPs at their surfaces without the participation of additional stabilizing ligands or polymers (see Scheme 1), leading to partially ligand-free metal surfaces.

**Figure 1:**
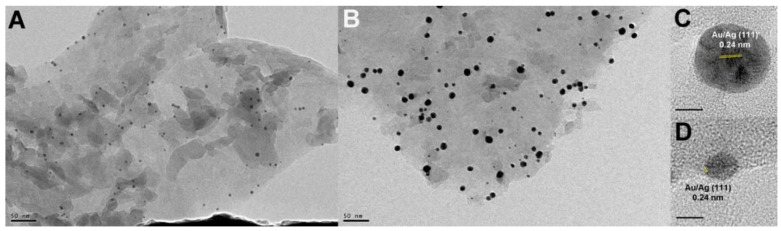
TEM images of AuAg-g-C_3_N_4_ nanohybrid **2** (**A**) and **3** (**B**). HRTEM images for ca. 10 nm size NPs in **3** (bar corresponds to 5 nm) (**C**) and ca. 3 nm size NPs in **3** (bar corresponds to 5 nm) (**D**).

Figure 1 depicts TEM images of the formed AuAg-g-C_3_N_4_ nanohybrids **2–3**. The low magnification images (Figure 1A,B) show the two homogeneous distributions of spherical NPs of 2.4 ± 1.1 nm and 5.0 ± 0.6 nm size for **2** and 3.0 ± 0.6 nm and 9.8 ± 0.6 nm size for **3** (see size histograms in Supplementary Materials), which are grafted on the surface of g-C_3_N_4_ nanosheets. The HRTEM images (Figure 1C,D) show polycrystalline nanoparticles of both sizes in **3** with the d spacing between lattice fringes of 0.24 nm that is assigned to (111) planes of gold or silver. In addition, the HRETM clearly shows the capping effect of g-C_3_N_4_ nanosheets that surround the nanoparticle.

**Figure 2:**
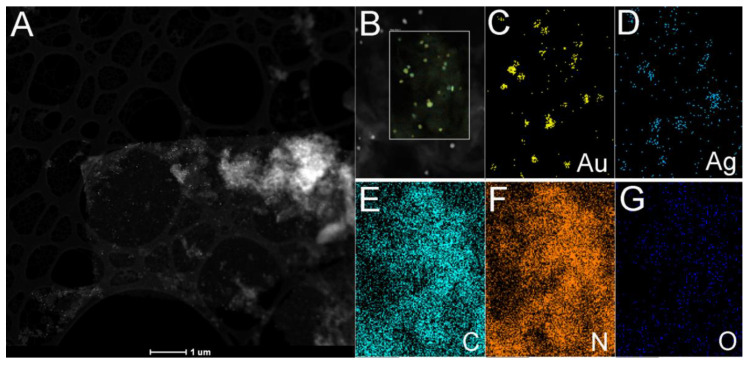
HAADF-STEM image of AuAg-g-C_3_N_4_ nanohybrid **3** (**A**) displaying a 2D sheet of g-C_3_N_4_ with metal nanoparticles grafted at the surface. EDS elemental mapping images for elements Au, Ag, C, N and O: image (**B**) displays all elements mapping at a time, while images (**C**–**G**) display the individual elemental maps.

The presence of two types of spherical NPs grafted at the surface of g-C_3_N_4_ prompted us to carry out an in-depth study of their composition through a HAADF-STEM/EDS study of a sample of AuAg-g-C_3_N_4_ nanohybrid **3**. Figure 2A shows HAADF-STEM image of **3** in which the 2D nature of g-C_3_N_4_ nanosheets is observed together with the presence of small size NPs with a high Z-contrast. The EDS analysis of a portion of the sample is depicted in Figure 2B–G.

The homogeneous distribution of N and C atoms along the whole sample points to the presence of g-C_3_N_4_ nanosheets. It is worth mentioning that oxygen is also homogeneously distributed over carbon nitride pointing to an interesting modification of its surface upon thermal treatment in the presence of ethylene glycol (vide infra). Also, interestingly, the distribution of Au and Ag along the sample does not match at the same positions, pointing to the formation of Au-enriched spherical NPs of ca. 10 nm size (yellow-mapped color in Figure 2C), whereas Ag atoms are more broadly distributed in the form of smaller size nanoparticles around the gold ones and over the whole carbon nitride surface (Figure 2D).

**Figure 3:**
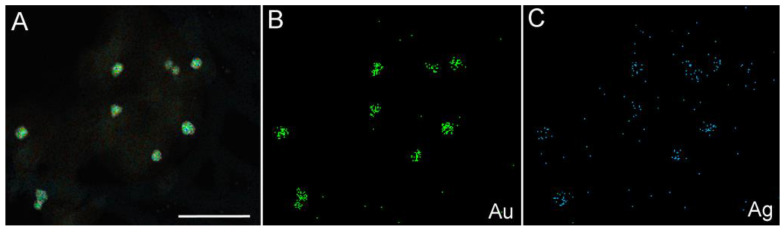
HAADF-STEM image of AuAg-g-C_3_N_4_ nanohybrid **3** displaying 10 nm size metal nanoparticles. EDS elemental mapping images for elements Au and Ag: image (**A**) displays both elements mapping at a time, while images (**B**,**C**) display the individual Au and Ag elemental maps, respectively (bar corresponds to 100 nm).

**Figure 4:**
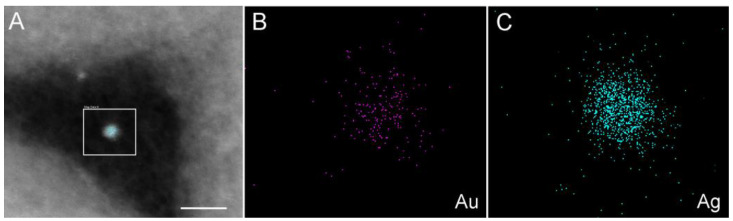
Detailed HAADF-STEM image of AuAg-g-C_3_N_4_ nanohybrid **3** displaying a 4 nm size metal nanoparticle. EDS elemental mapping images for elements Au and Ag: image (**A**) displays both elements mapping at a time, while images (**B**,**C**) display the individual Au and Ag elemental maps, respectively (bar corresponds to 10 nm).

An even more detailed analysis is shown in Figure 3 and Figure 4 and in Table S1 and Figures S3–S7 in Supplementary Materials. As it can be observed, the EDS mapped analysis of the composition of ca. 10 nm size Au-Ag NPs in Figure 3 indicates a main gold composition, whereas the same analysis for ca. 3 nm size Au-Ag NP indicates a main silver composition (Figure 4). This analysis has been repeated for several nanoparticles. The low oxygen concentration found around the small Ag-enriched nanoparticles rules out the formation of Ag_2_O or AgO NPs (see Supplementary Materials). Therefore, this analysis confirms the presence of bimetallic AuAg alloyed nanoparticles displaying two distinct sizes and compositions. The larger ca. 10 nm size NPs are gold-enriched, meanwhile the smaller 4 nm size NPs are silver enriched alloy NPs, respectively.

**Figure 5:**
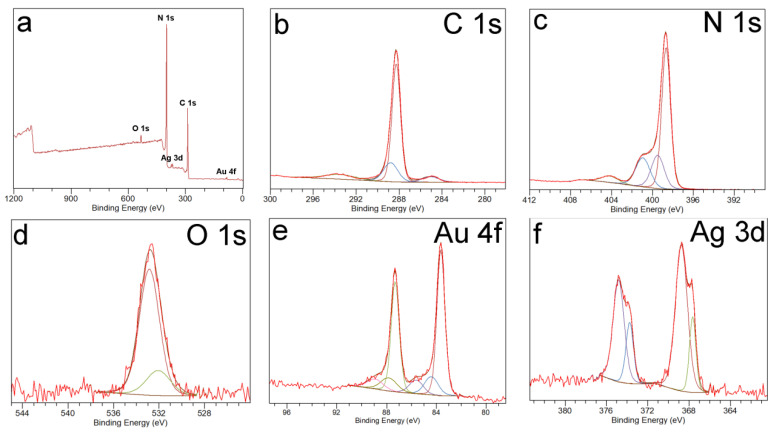
Wide XPS spectrum for AuAg-g-C_3_N_4_ **3** nanohybrid (**a**). High-resolution XPS spectra for C 1s (**b**), N 1s (**c**), O 1s (**d**), Au 4f (**e**) and Ag 3d (**f**).

XPS analysis was carried out in order to gain insight into the surface elemental composition and the elemental chemical states of the atoms forming AuAg-g-C_3_N_4_ nanohybrids **2**–**3** (see Figure 5 for **3** and Supplementary Materials for pristine g-C_3_N_4_ **1** and nanohybrid **2**).

The wide XPS spectra display the presence of intense peaks corresponding to C and N, meanwhile small peaks assigned to O, Au and Ag can also be detected. From the analysis of these wide spectra, it can be observed that the wt% composition of Ag and Au atoms is 0.9 and 0.9% (2) and 1.7 and 1.3% (3), respectively, pointing to some loss of g-C_3_N_4_ in the experimental workup, since a 0.5 wt% (2) or 1 wt% (3) of metals (gold + silver) was added in the preparation of nanohybrids **2** and **3**. It is also worth mentioning that the amount of oxygen (1.8 wt% (2) or 2.3 wt% (3)) is larger than the one obtained for pristine g-C_3_N_4_ **1** (0.78 wt%) pointing to an enrichment of oxygen species at the surface of this semiconductor, probably due to the synthetic thermal conditions in ethylene glycol. This result is similar to a previously reported one, in which the microwave treatment of g-C_3_N_4_ in ethanol led to an increased density of oxygen-containing species (-OH groups) at the g-C_3_N_4_ surface, conferring a higher hydrophilicity, better charge carrier separation and pollutant adsorbance [26]. In our case the presence of an increased amount of hydroxyl groups in AuAg-g-C_3_N_4_ nanohybrids **2**–**3** would favor the charge carrier separation.

Figure 5b depicts the high-resolution XPS spectrum for the C 1s region for nanohybrid **3**, which is fitted into four peaks. The intense peak at 288.3 eV is assigned to the sp^2^ carbon atoms in the triazine units of g-C_3_N_4_ (C − N = C), supported by the presence of the weak peak component of the C 1s region at 293.6 assigned to double bond π-excitations. The peak at 288.8 eV would be related to C-O bonds, meanwhile the peak at 285.1 is associated to C-C bonds [27,28]. In addition, Figure 5c provides the high-resolution spectrum of the N 1s region fitted again into four peaks. In a similar way, the intense peak at 398.6 eV can be associated to sp^2^ N atoms in the triazine (−C−N=C−) unit, again supported by the presence of a weak peak at 404.1 eV arising from the double bond π-excitations. The N atoms between aromatic rings in the C_3_N_4_ structure is assigned to the weak peak at 399.5 eV, whereas the presence of NH_2_ groups is related to the peak at 400.9 eV [27,28]. The assignment of the C 1s and N 1s regions for AuAg-g-C_3_N_4_ nanohybrid **3** is very similar to the one obtained for pristine g-C_3_N_4_ **1** and nanohybrid **2** (see Supplementary Materials). On the other hand, the high-resolution XPS spectrum for the O 1s region (Figure 5d) displays a peak at 532.4 eV fitted into two peaks attributed to the presence of hydroxyl groups at the semiconductor surface (531.6 eV) and to adsorbed water molecules (532.6 eV). The O 1s peak value is similar to the peak obtained for a previously reported Au-g-C_3_N_4_ nanohybrid at ca. 532 eV [29] and also similar to the one obtained for pristine g-C_3_N_4_ **1** and nanohybrid **2** (see Supplementary Materials).

The high-resolution XPS spectrum for the Au 4f region of **3** is shown in Figure 5e. The experimental signals are fitted to three spin-orbit doublets of different intensity, but equally separated in energy (ca. 3.7 eV). The most intense doublet at 83.6 eV and 87.3 eV can be assigned to metallic gold; the less intense doublets can be attributed to the presence of Au(I) and Au(III) oxidation states. The presence of gold atoms in positive oxidation states is attributed to a higher fraction of surface atoms with low coordination numbers when small size (sub-10 nm) nanoparticles are grafted on the carbon nitride surface [29,30]. In addition, the assignment of the Au(I) or Au(III) peaks to the organometallic precursor is ruled out since the presence of fluorine atom peaks arising from the pentafluorophenyl ligands are not observed.

Similarly, the high resolution XPS spectrum for the Ag 3d region of **3** displayed in Figure 5f shows two experimental signals, which are fitted to two spin-orbit doublets with a separation in energy of ca. 6.0 eV, characteristic of silver 3d region. Taking into account the energy maxima of the doublet with peaks at 368.7 and 374.8 eV, we can assign this high intensity signals to metallic silver. The signals at 367.6 and 373.7 at slightly lower energy are related to the presence of Ag(I) due to the same reason as the one discussed for gold, i.e., the small size of silver NPs leads to a higher fraction of surface silver atoms with low coordination numbers [31].

**Figure 6:**
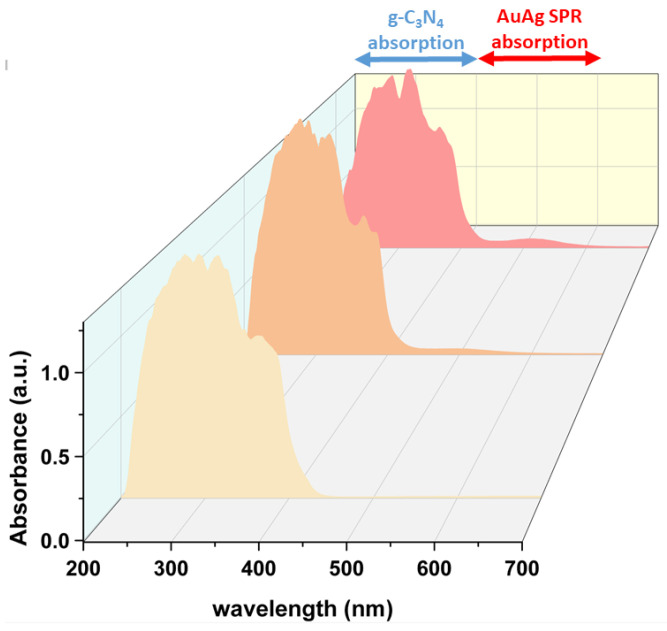
Solid UV-vis spectra for pristine g-C_3_N_4_ **1** (beige) and AuAg-g-C_3_N_4_ nanohybrids **2** (orange) and **3** (red).

The presence of Au-Ag NPs of different sizes and alloy compositions over g-C_3_N_4_ provides an interesting material with a wide plasmonic absorption in the visible range arising from the variable metal composition of the grafted plasmonic nanoparticles. The objective pursued with the inclusion of Au-Ag NPs on carbon nitride nanosheets consists of enhancing the photocatalytic performance of this material under visible light. In this sense, the solid-state UV-vis analysis of AuAg-g-C_3_N_4_ nanohybrids **2** and **3** provides interesting insights (see Figure 6). Thus, the inclusion of plasmonic Au-Ag NPs extends the visible light absorption from 400 to 600 nm, through the appearance of the expected plasmonic absorption of the alloyed NPs. The plasmonic absorption arising from pure spherical Ag NPs in solution appears in the 400–420 nm range [32], whereas the plasmonic absorption arising from pure Au NPs in solution appears around 520–530 nm [12]. In the present case the two types of alloyed compositions found, i.e., silver and gold-enriched, permits to obtain a broad plasmonic absorption taking into account that the small size silver enriched NPs will absorb the higher energies of the visible light (ca. 400–420 nm) and the gold enriched ones will display a maximum around 520 nm, absorbing intermediate energies of the visible light. Also, the heterojunction between the bimetallic NPs and g-C_3_N_4_ produces a slight decrease of the g-C_3_N_4_ band gap from 2.74 to 2.71 (**2**) or 2.69 eV (**3**) (see Tauc plots in Supplementary Materials).

### 3.2. Mechanism of Formation of Photocatalysts

The use of organometallic compounds as precursors constitutes a very interesting approach for the synthesis of metallic nanostructures. In the case of pentafluorophenyl derivatives of gold (I) and/or silver (I), the reduction of metals under very mild reaction conditions has allowed the preparation of new types of nanostructures such as ultra-fine nanorods or nanowires [33], ultra-small gold nanoparticles [34] or gold colloidosomes [35]. There are usually two decomposition mechanisms for these compounds, i.e., bimolecular reductive elimination or deprotonation of acidic ligands by means of the basic pentafluorophenyl ligand. In the first case, the pentafluorophenyl ligands act as reducing agents by self-oxidizing to decafluorobiphenyl species, easily detectable in solution by ^19^F nuclear magnetic resonance. In the study of the behaviour of the [Au_2_Ag_2_(C_6_F_5_)_4_(OEt_2_)_2_]_n_ precursor in the presence of g-C_3_N_4_ we have observed that the decomposition of the precursor occurs through the bimolecular reductive elimination mechanism (see Supplementary Materials). In addition, it is observed through ^19^F-NMR, that during the course of the decomposition reaction, the only fluorinated species that remains in solution is the anion [Au(C_6_F_5_)_2_]^−^, which indicates that in the reaction medium (ethyleneglycol) the organometallic precursor dissociates into its ionic components [Au(C_6_F_5_)_2_]^−^ and [Ag(OEt_2_)]^+^, as expected when donor solvents are used (see Supplementary Materials).

Once the molecular mechanism of decomposition of the organometallic precursor is elucidated, the most striking result is the presence of two distinct populations of metal NPs in terms of size (2.4 nm vs. 5.0 nm in **2** or 3.0 nm vs. 9.8 nm in **3**) and composition (silver-enriched vs. gold-enriched). At this regard, the previous study by some of us of the decomposition of complex [Au_2_Ag_2_(C_6_F_5_)_4_(OEt_2_)_2_]_n_ in the presence of hexadecylamine (HDA) showed the formation in solution of neutral [M(C_6_F_5_)(HDA)] (M = Au, Ag) species [22]. In addition, the decomposition of such intermediates displayed different reduction rates, allowing the control of the formation of AuAg alloyed nanoparticles with different metal compositions, depending on the experimental conditions. This type of species would also be formed at some points of the surface of g-C_3_N_4_ through the coordination of the [M(C_6_F_5_)] units to the triazine moieties of g-C_3_N_4_ or, alternatively, Ag(I) centres would coordinate to N-donor atoms of triazine units leading to ionic [Ag(triazine)][Au(C_6_F_5_)_2_] species acting as nucleation points for nanoparticle growth. Unfortunately nor the neutral neither the ionic species have been detected, although the equimolecular reaction between complex [Au_2_Ag_2_(C_6_F_5_)_4_(OEt_2_)_2_]_n_ and triazine as ligand gives rise to the formation of the ionic species in solution (see Supplementary Materials), in contrast to the reactivity of HDA towards the same complex.

**Figure 7:**
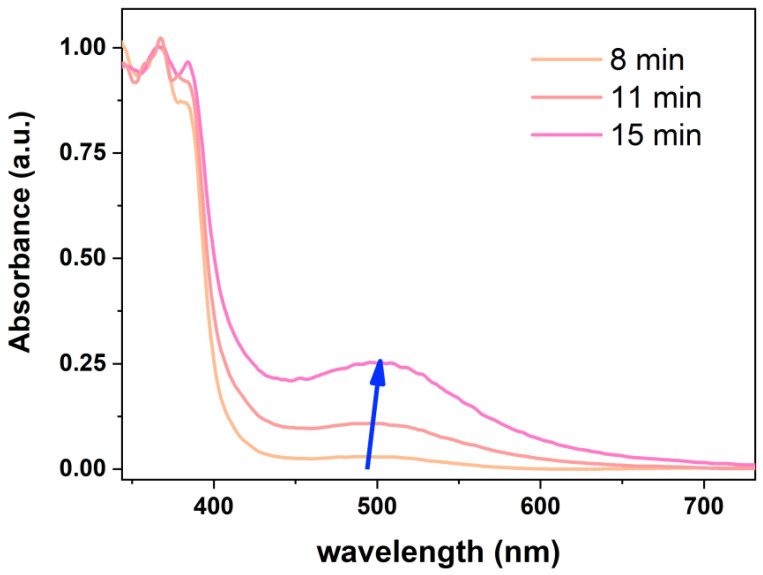
Time-monitored solid UV-vis spectra displaying the plasmonic absorption in the formation process of AuAg-g-C_3_N_4_ nanohybrid (**3**). The blue arrow indicates a slight red-shift of the plasmonic absorption with time.

In any case, from the earlier studies we could anticipate that the Ag(I) intermediates are less stable and easier to reduce, leading to initial formation of Ag NPs. This observation was time-monitored through UV-vis absorption spectroscopy for HDA-capped AuAg alloyed NPs [22]. In the present study, we have collected the solid UV-vis spectrum at different reaction times for the process of formation of AuAg-g-C_3_N_4_ nanohybrid (**3**). Although the plasmon band shift in solid samples is not as evident as in solution, the solid UV-vis spectra display a slight red-shift of the plasmon absorption band with time in agreement with a sequential growth of plasmonic nanoparticles of different compositions (see Figure 7). Both the possible formation of distinct nucleation points of Au(I) and Ag(I) and the faster rate of formation of small Ag NPs at the surface of g-C_3_N_4_ would support the possibility of a separate growth of two distinct types of metal nanoparticles. Based on the analysis of this mechanism a further control of the reaction conditions, specially using lower temperatures, would provide the possibility of obtaining composition-controlled populations of Au-Ag NPs on g-C_3_N_4_ surfaces mostly enriched with one of the metals.

### 3.3. Photodegradation of Ibuprofen

The synthesis of AuAg-g-C_3_N_4_ nanohybrids displaying broad plasmonic absorptions thanks to the formation of two types of plasmonic NPs at the surface of g-C_3_N_4_, prompted us to study their photocatalytic behavior under visible light towards the degradation of ibuprofen.

**Figure 8:**
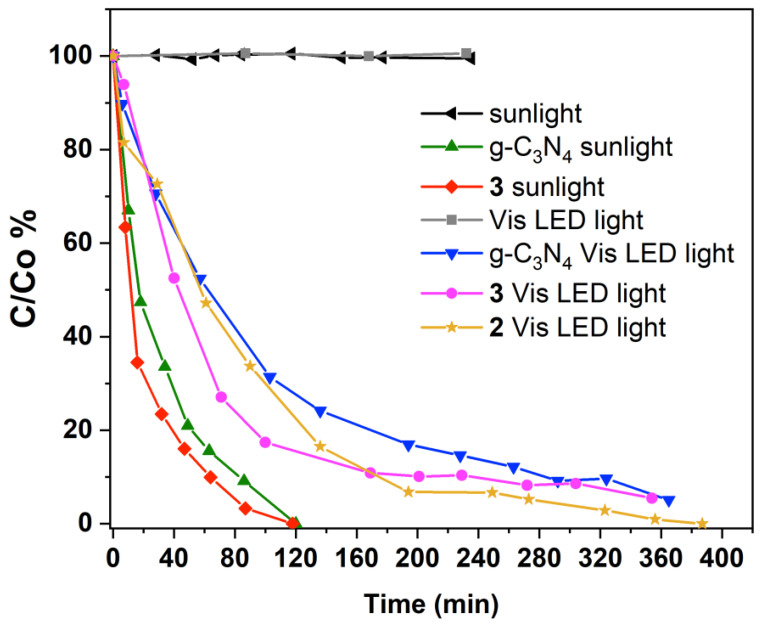
Evolution of ibuprofen concentration during its degradation with g-C_3_N_4_ and nanohybrids **2** and **3**, under visible LED light or under sunlight.

Figure 8 compares the photocatalytic degradation of ibuprofen using pristine g-C_3_N_4_ or AuAg-g-C_3_N_4_ nanohybrids **2** and **3** under low power visible LED light source. Pristine g-C_3_N_4_ **1** and nanohybrid **3** were also tested using sunlight. The corresponding blank experiments (photolysis) are also included in Figure 8. The photolysis depletion of ibuprofen with both sources of light was negligible, which illustrates the low photodegradability and persistence of ibuprofen in water. The degradation rates observed in the experiments performed under sunlight were faster than under LED light. The ibuprofen sample was completely removed within 120 min under sunlight, whereas around 5% of ibuprofen remained in solution after 360 min when the mixtures were irradiated with LED light. On the other hand, when the catalyst employed in the degradations contained nanoparticles of noble metals (gold and silver), the pseudo-first-kinetic constants obtained were larger than with g-C_3_N_4_. The kinetic constants obtained in the photodegradations with g-C_3_N_4_ and AuAg-g-C_3_N_4_ nanohybrid **3** under sunlight were 0.027 and 0.036 min^−1^; g-C_3_N_4_, AuAg-g-C_3_N_4_ nanohybrid **2** and AuAg-g-C_3_N_4_ nanohybrid **3** under visible LED light were 0.0086, 0.012 and 0.012 min^−1^, respectively (see Supplementary Materials).

The presence of two types of metal nanoparticles leading to a broad plasmonic absorption in nanohybrids **2** and **3** clearly improved the efficiency of the photodegradations with respect to g-C_3_N_4_. In general, the formation of metal-semiconductor heterojunctions through the grafting of metal NPs at the surface of a semiconductor would increase the photocatalytic efficiency through several paths: (i) the metal NP may act as co-catalyst; (ii) the metal NPs can improve the electron-hole separation acting as electron sinks thanks to the formation of a Schottky barrier between the metal and the semiconductor; and (iii) when the metal NPs display plasmonic properties, the localized surface plasmon resonance (LSPR) induces additional local temperature increase (improves exciton formation in the semiconductor), optical near field enhancement (transfer of photons to the semiconductor) and hot-electron injection from the plasmonic nanoparticle to the conduction band of the semiconductor, preventing a fast electron-hole recombination [10,11,19].

**Figure 9:**
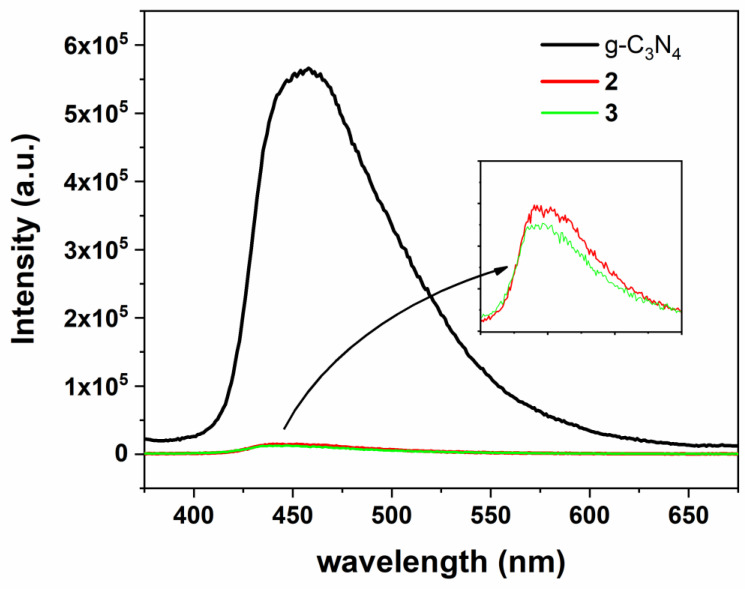
Photoluminescent spectra of g-C_3_N_4_ and nanohybrids **2** and **3** in solid state (excitation wavelength is 350 nm).

An indirect proof of the improved charge separation is the analysis of the photoluminescent (PL) properties of g-C_3_N_4_ based materials. Figure 9 depicts the emission spectrum of pristine g-C_3_N_4_ with a maximum at 440 nm (excitation at 350 nm). This emission band arises from interband recombination of the photogenerated electron-hole pairs being the energy of the emission the one corresponding to the semiconductor band-gap. The formation of plasmonic AuAg NPs of two distinct populations of Ag-enriched and Au-enriched compositions at the surface of g-C_3_N_4_ provokes a drastic quenching of the photoemissive properties of nanohybrids **2** and **3**, suggesting a greatly improved and efficient electron-hole separation for these systems. This result would be in agreement with an effective electron-trapping effect by the Au-Ag NPs arising from the formation of the Schottky barrier, i.e., heterojunction effect, although favorable LSPR effects may also contribute to the improved photocatalytic properties.

**Figure 10:**
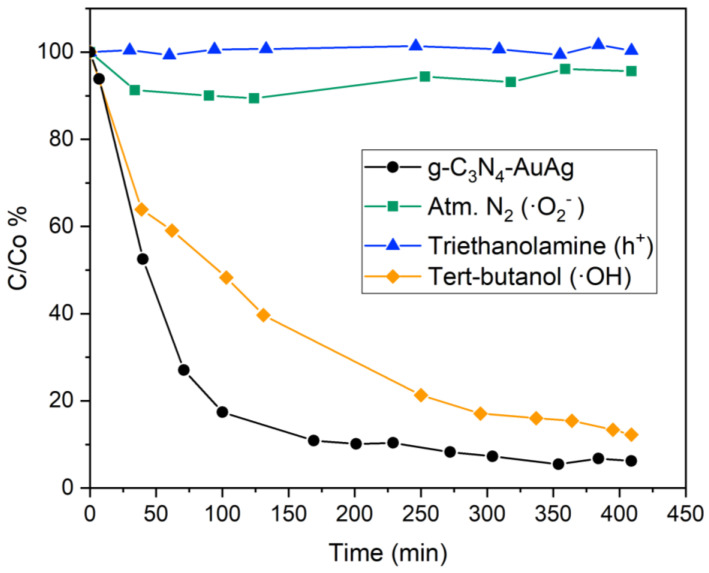
Evolution of ibuprofen concentration during photocatalytic degradation with **3** under visible light irradiation and effect of scavengers.

**Figure 11:**
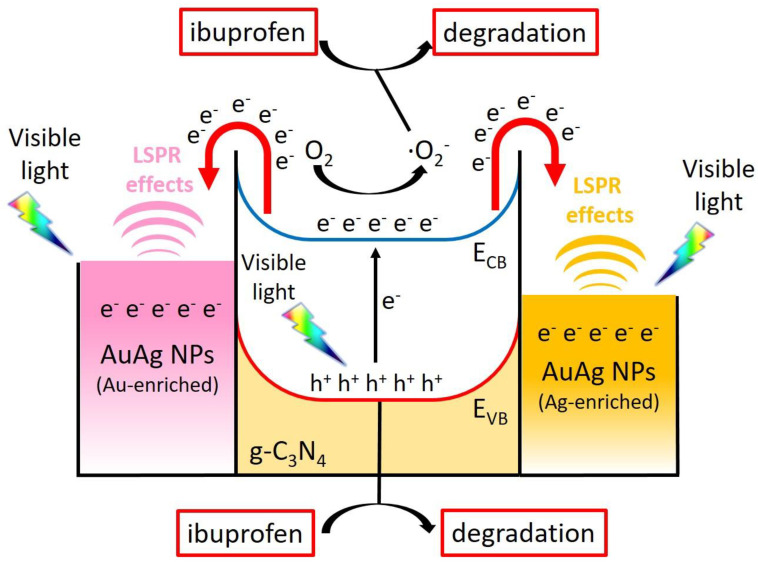
Schematic representation of the mechanism of degradation of ibuprofen through the plasmonic hot-electron injection from Au-enriched and Ag-enriched NPs and from electronic excitation of g-C_3_N_4_ semiconductor.

In order to elucidate the mechanism of degradation of ibuprofen, quenching experiments for the detection of reactive oxygen species were performed. During the photocatalytic degradation process, many reactive oxygen species responsible for pollutants’ depletion can be generated. To evaluate which species played a main role in the degradation process of ibuprofen under visible light, catalyzed by AuAg-g-C_3_N_4_ nanohybrid **3** and irradiation, three trapping experiments were carried out. *tert*-Butanol was used as scavenger for hydroxyl radicals (·OH). Triethanolamine was employed as scavenger for photogenerated holes (h^+^). In addition, to study the influence of superoxide radicals (·O_2_^−^), the degradation was performed under inert N_2_ atmosphere to quench them [36,37,38,39]. As can be seen in Figure 10, triethanolamine completely quenched the ibuprofen degradation and when the reaction was evaluated under N_2_ atmosphere the degradation rate was also greatly quenched. In contrast, *tert*-butanol had a slight effect on the photodegradation. According to these results, the holes (h^+^) and superoxide radicals (·O_2_^−^) are the main species involved in the photocatalysis, whereas the hydroxyl radicals (·OH) play a minor role in the photocatalytic mechanism. Taking into account scavenger effects, it is likely that under visible light irradiation both heterojunction effects and LSPR effects play an important role. On the one hand, the drastic charge carrier separation found for nanohybrids **2** and **3** agrees with a migration of photogenerated electrons in the g-C_3_N_4_ semiconductor towards Au-Ag NPs. In addition, LSPR effects such as localized heating or near-field enhancement would also assist the charge carrier separation at the semiconductor. The transfer of plasmonic hot electrons from Ag-enriched and Au-enriched NPs to the CB g-C_3_N_4_ would also provide a charge carrier separation at the plasmonic NPs. In any case, the obtained charge carrier separation greatly favors the formation of the superoxide radicals (·O_2_^−^) through the reduction of oxygen thanks to the photogenerated electrons at the CB of g-C_3_N_4_ or Au-Ag NPs. Indeed, according to previous reports [40,41], the CB edge of g-C_3_N_4_ is more negative (−1.19 eV, vs. NHE) than the O_2_/·O_2_^−^ potential (−0.33 eV, vs. NHE). At the same time, holes (h^+^) are formed at the plasmonic NPs and at the VB of g-C_3_N_4_. Therefore, in addition to the superoxide radicals (·O_2_^−^) the holes (h^+^) generated also participate in the degradation of ibuprofen, in agreement with the experimental findings (see Figure 11).

The secondary role played by hydroxyl radicals is in agreement with the unfavourable potential of the g-C_3_N_4_ VB (1.65 eV, vs. NHE) for the formation of the OH radicals from OH^−^ groups (OH/OH^−^ potential is 2.38 eV, vs. NHE) or from H_2_O (OH/H_2_O potential is 2.72 eV, vs. NHE). In this case, the ·OH radical species might be formed in an indirect manner from H_2_O_2_, because of the following reduction pathway [42]:

g-C_3_N_4_ (e^−^) + O_2_ → ·O_2_^−^

g-C_3_N_4_ (e^−^) + ·O_2_^−^ + 2H^+^ → H_2_O_2_

g-C_3_N_4_ (e^−^) + H_2_O_2_ → ·OH + OH^−^

Finally, the mechanism of degradation of ibuprofen under sunlight would be similar since both the hot-electron injection in the CB of g-C_3_N_4_ and the electronic excitation from the VB to the CB of the g-C_3_N_4_ semiconductor could be reached. The larger irradiance of the sunlight with respect to visible LED light would be responsible for the faster kinetics of the processes involved thanks to the heterojunction and LSPR effects.

## 4. Conclusions

We have developed a facile approach for the synthesis of Au-Ag/g-C_3_N_4_ nanohybrids with enhanced photocatalytic activity in degradation of ibuprofen under white LED light and under sunlight. The in-depth analysis of the nanohybrids composition points to the formation of two types of alloyed Au-Ag NPs differing in terms of size and composition in a single synthetic step. Thus, small size Ag-enriched NPs and larger size Au-enriched NPs provide a broad plasmonic absorption in the visible that, together with heterojunction effects greatly preclude the electron-hole recombination at the semiconductor. This improved charge-carrier separation enhances the photocatalytic efficiency of the nanohybrid photocatalysts towards the degradation of ibuprofen, which is a persistent drug in water. Quenching experiments have provided the information for proposing a mechanism of photodegradation of ibuprofen using Au-Ag/g-C_3_N_4_ nanohybrid as photocatalyst. Superoxide radicals and holes are the main active species involved in the degradation process. More studies based on the use of real polluted water samples and up-scaling of the photocatalytic process are underway.

## Data Availability

Data sharing is not applicable.

## Supplementary Materials

The following are available online at https://www.mdpi.com/article/10.3390/ma14143912/s1, Figure S1. Size histograms of Ag-enricbed AuAg NPs (left) and Au-enriched AuAg-NPs (right) on nanohybrid **2**, Figure S2. Size histograms of Ag-enricbed AuAg NPs (left) and Au-enriched AuAg-NPs (right) on nanohybrid **3**, Figure S3. HAADF-STEM images of AuAg-g-C_3_N_4_ nanohybrid **3** (A-B) displaying Au-enriched AuAg nanoparticles grafted on the surface of g-C_3_N_4_ and EDS individual elemental mappings for elements Au, Ag, C, N and O. Note that the HAADF-STEM images A-B display all elements mapping at a time, Figure S4. EDS spectra of selected Au-enriched Au-Ag NPs of ca. 10 nm size (NPs **1** and **2** in Table S1), Figure S5. EDS spectra of selected Ag-enriched Au-Ag NPs of ca. 5 nm size. (NPs **3**–**5** in Table S1), Figure S6. EDS spectra of selected Ag-enriched Au-Ag NPs of ca. 5 nm size. (NPs **6**–**10** in Table S1), Figure S7. EDS spectra of selected Ag-enriched Au-Ag NPs of ca. 5 nm size. (NPs **11**–**17** in Table S1), Figure S8. HAADF-STEM images of AuAg-g-C_3_N_4_ nanohybrid **3** (A-D) displaying Ag-enriched AuAg nanoparticles of ca. 5 nm size grafted on the surface of g-C_3_N_4_ and EDS individual elemental mappings for elements Au, Ag and O. Note that the HAADF-STEM images A-D display all elements mapping at a time. The analysis of the oxygen content discards the presence of Ag_2_O species (bars correspond to 10 nm), Figure S9. Wide XPS spectrum for pristine g-C_3_N_4_ (a). High-resolution XPS spectra for O 1s (b) C 1s (c), N 1s (d), Figure S10. Wide XPS spectrum for AuAg- g-C_3_N_4_ nanohybrid **2** (a). High-resolution XPS spectra for C 1s (b) N 1s (c), O 1s (d), Au 4f (e), Ag 3d (f), Figure S11. Tauc plots for **1**–**3**, Figure S12. ^19^F NMR spectrum of the in situ formation of AuAg-g-C_3_N_4_ nanohybrid 3. The obtained profile is assigned to the presence in solution of the molecule C_6_F_5_-C_6_F_5_ as a byproduct of the reduction of Au(I) and Ag(I) ions to the zeroth oxidation state., Figure S13. ^19^F NMR spectrum of the reaction mixture of complex [Au_2_Ag_2_(C_6_F_5_)_4_(OEt_2_)_2_]_n_ and g-C_3_N_4_. The presence of broad signals assigned to [Au(C_6_F_5_)_2_]^−^ units in solution agrees with the dissociation of the precursor into its ionic components [Au(C_6_F_5_)_2_]^−^ and [Ag(OEt_2_)]^+^, as expected when a donor solvent such as ethyleneglycol is used, Figure S14. ^19^F NMR spectrum of the in situ reaction between complex [Au_2_Ag_2_(C_6_F_5_)_4_(OEt_2_)_2_]_n_ in the presence of melamine in a 4:2 metals:melamine ratio. The obtained profile is assigned to the presence of [Au(C_6_F_5_)_2_]^−^ units in solution, in agreement with the coordination of silver to melamine, Figure S15. Kinetics of the photocatalysts **1** (left), **2** (center) and **3** (right) under visible light. The fitting results are represented assuming a pseudo-first order reaction, Figure S16. Kinetics of the photocatalysts **1** (left), and **3** (right) under sunlight. The fitting results are represented assuming a pseudo-first order reaction. Table S1. EDS analysis of AuAg NPs on nanohybrid **3** showing the wt% composition of Au, Ag and O.
