# Supplementary material for: Combination of Au-Ag Plasmonic Nanoparticles of Varied Compositions with Carbon Nitride for Enhanced Photocatalytic Degradation of Ibuprofen under Visible Light
Source: Materials (Basel). 2021 Jul 14;14(14):3912. doi: 10.3390/ma14143912 (PMC8303281; doi:10.3390/ma14143912)
Supplement: Supplementary file 1 [file materials-14-03912-s001.zip › materials-1267654-supplementary.pdf]

materials

Supplementary Materials

Combination of Au-Ag Plasmonic Nanoparticles of Varied Compositions with Carbon Nitride for Enhanced Photocatalytic Degradation of Ibuprofen under Visible Light

Marta Jiménez-Salcedo, Miguel Monge * and María Teresa Tena

Centro de Investigación en Síntesis Química (CISQ), Department of Chemistry, University of La Rioja, Complejo Científico-Tecnológico, 26006 Logrono, Spain; marta.jimenez@unirioja.es (M.J.-S.); maria-teresa.tena@unirioja.es (M.T.T.)
* Correspondence: miguel.monge@unirioja.es

Citation: Jiménez-Salcedo, M.; Monge, M.; Tena, M.T. Combination of Au-Ag Plasmonic Nanoparticles of Varied Compositions with Carbon Nitride for Enhanced Photocatalytic Degradation of Ibuprofen under Visible Light. *Materials* **2021**, *14*, 3912. https://doi.org/10.3390/ma14143912

Academic Editor: Arunas Ramanavicius

Received: 3 June 2021
Accepted: 8 July 2021
Published: 14 July 2021

Publisher's Note: MDPI stays neutral with regard to jurisdictional claims in published maps and institutional affiliations.

Copyright: © 2021 by the authors. Licensee MDPI, Basel, Switzerland. This article is an open access article distributed under the terms and conditions of the Creative Commons Attribution (CC BY) license (http://creativecommons.org/licenses/by/4.0/).

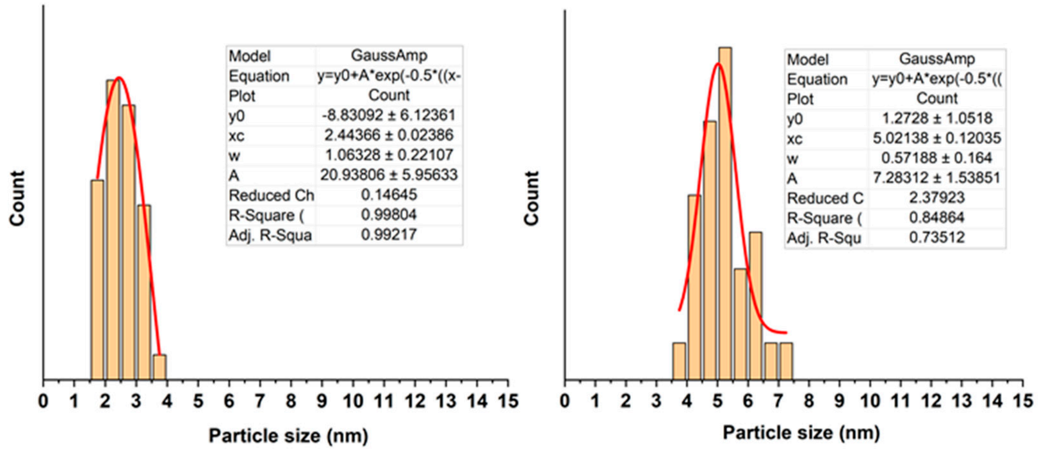

Figure S1. Size histograms of Ag-enricbed AuAg NPs (left) and Au-enriched AuAg-NPs (right) on nanohybrid **2.**

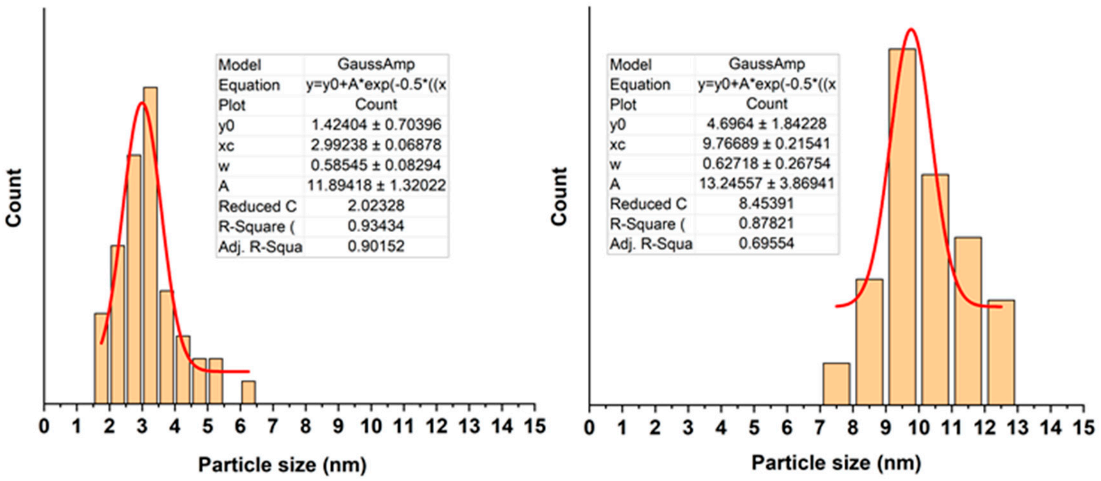

Figure S2. Size histograms of Ag-enricbed AuAg NPs (left) and Au-enriched AuAg-NPs (right) on nanohybrid **3.**

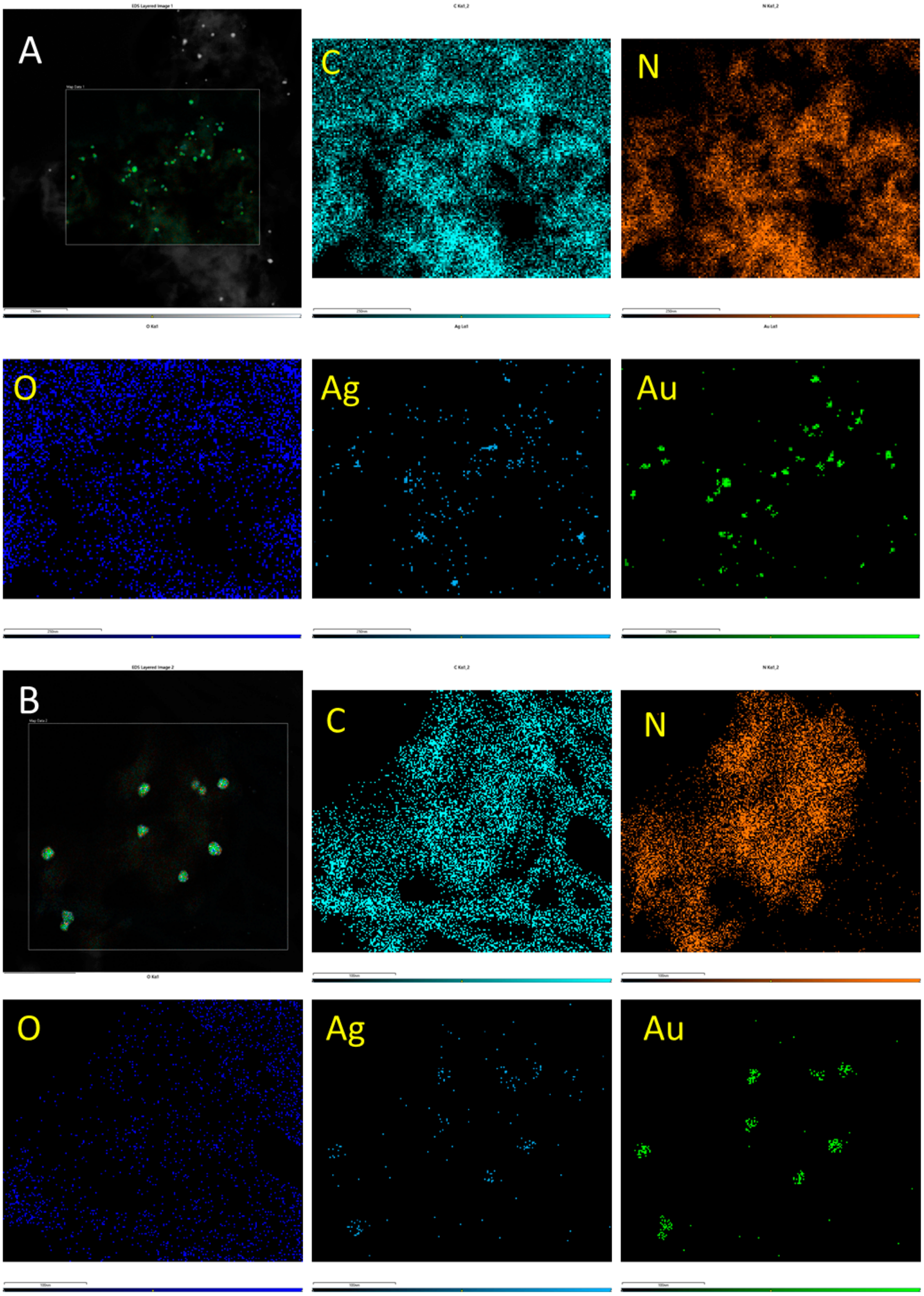

Figure S3. HAADF-STEM images of AuAg-g-C_3N_4 nanohybrid **3** (A-B) displaying Au-enriched AuAg nanoparticles grafted on the surface of g-C_3N_4 and EDS individual elemental mappings for elements Au, Ag, C, N and O. Note that the HAADF-STEM images A-B display all elements mapping at a time.

Table S1. EDS analysis of AuAg NPs on nanohybrid **3** showing the % wt composition of Au, Ag and O.

NP	Aprox Size (nm)	Au % wt	Ag % wt	O % wt
1	11	>90	<5	<5
2	11	>90	<5	<5
3	6.2	<5	>90	-
4	4.5	30	70	-
5	4.5	15	75	10
6	2.8	-	85	15
7	1.8	35	65	-
8	5.4	>10	85	-
9	3.6	>15	80	-
10	2.7	>10	85	-
11	3.3	>5	85	>5
12	4.7	>5	90	-
13	4.7	>5	90	-
14	3.1	<5	>90	<5
15	4.4	<5	>90	<5
16	2.5	<5	>90	<5
17	3.7	<5	>90	<5

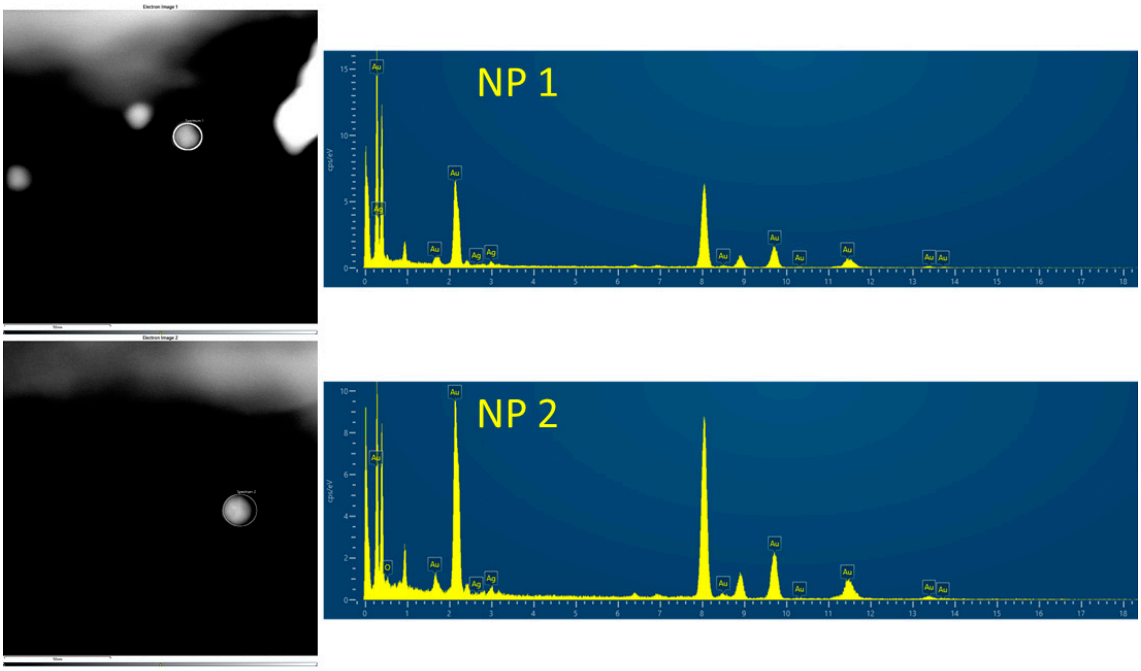

Figure S4. EDS spectra of selected Au-enriched Au-Ag NPs of ca. 10 nm size (NPs 1 and 2 in Table S1).

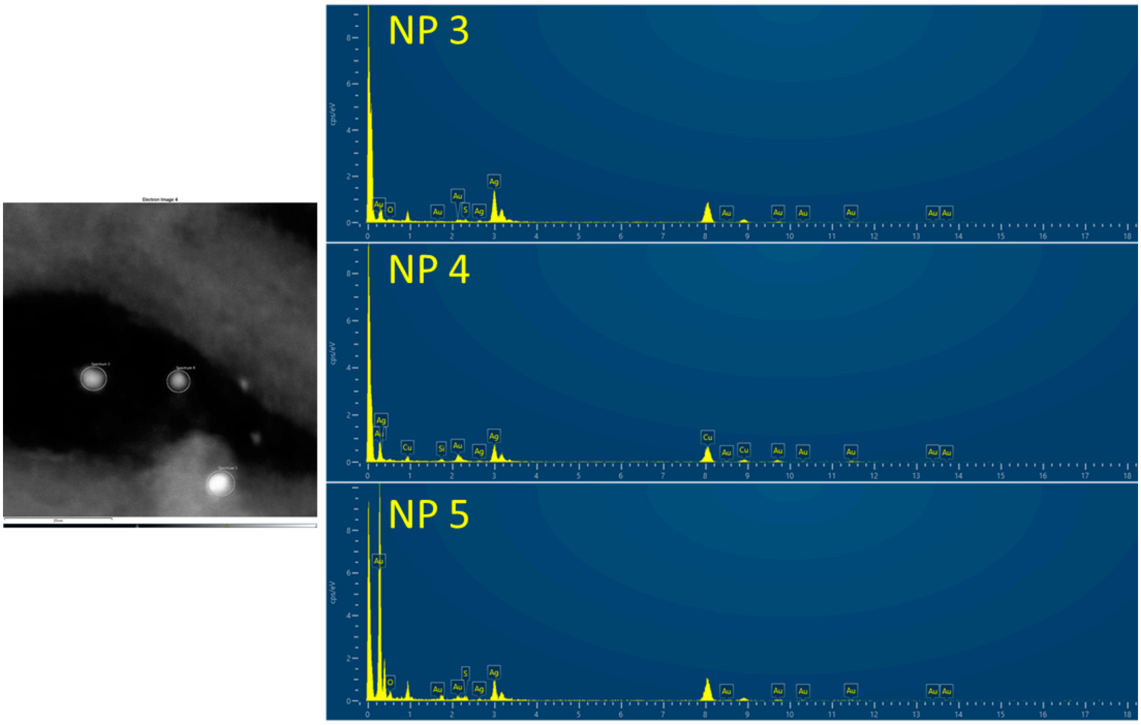

Figure S5. EDS spectra of selected Ag-enriched Au-Ag NPs of ca. 5 nm size. (NPs 3–5 in Table S1).

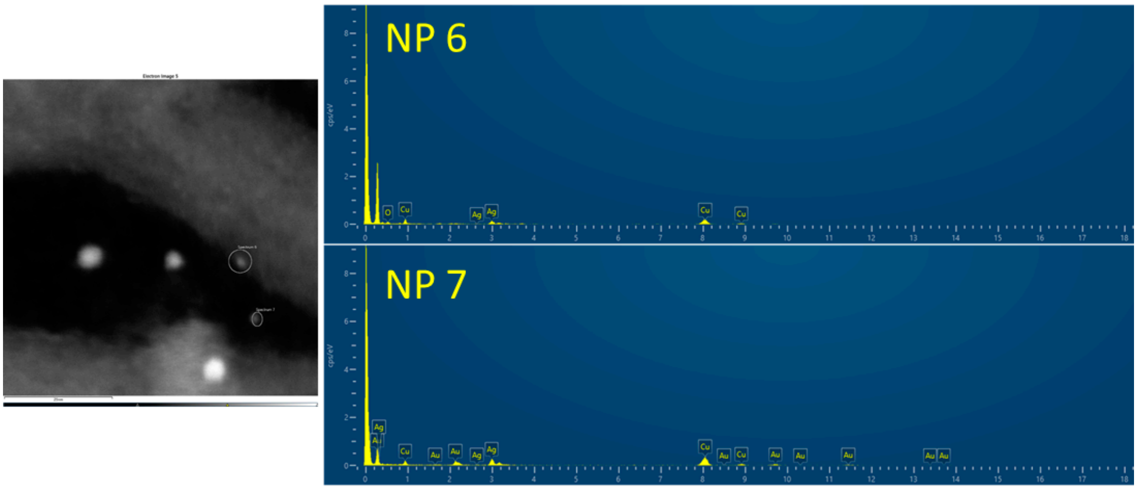

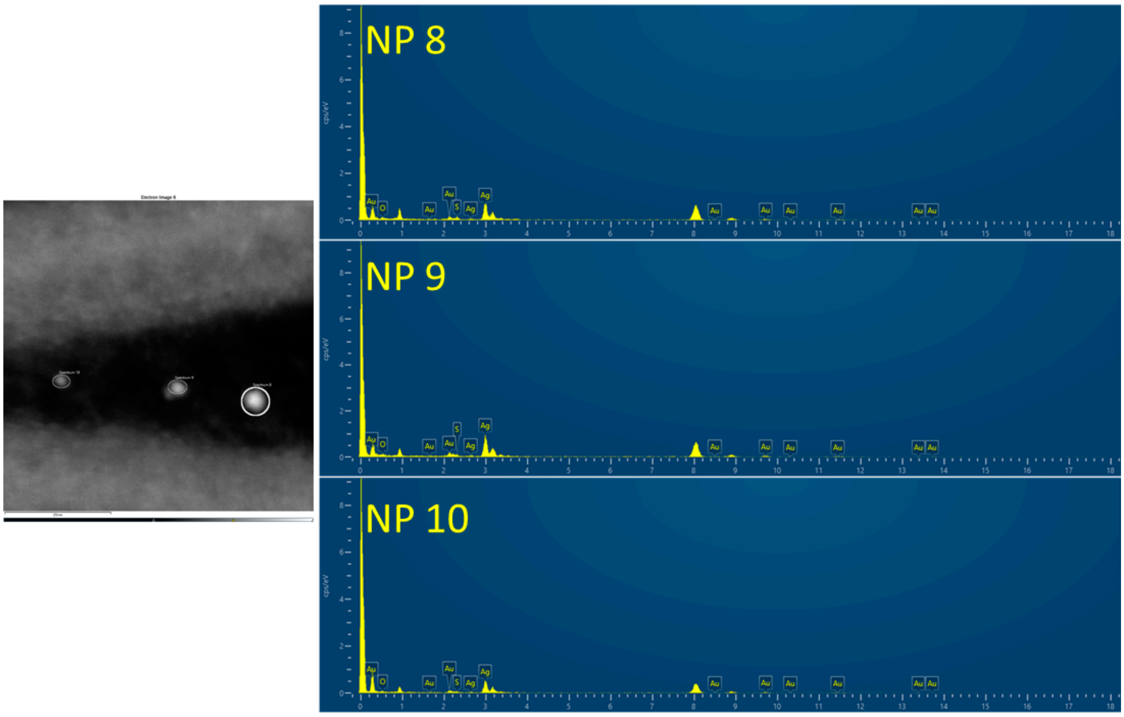

Figure S6. EDS spectra of selected Ag-enriched Au-Ag NPs of ca. 5 nm size. (NPs 6–10 in Table S1).

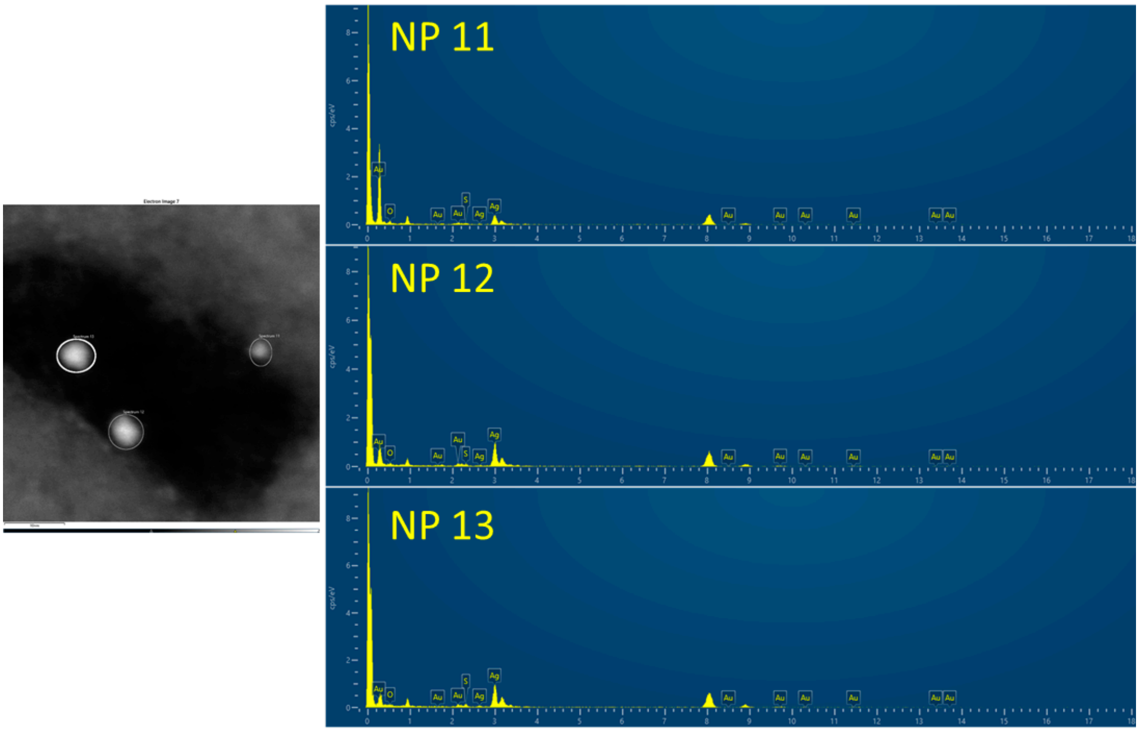

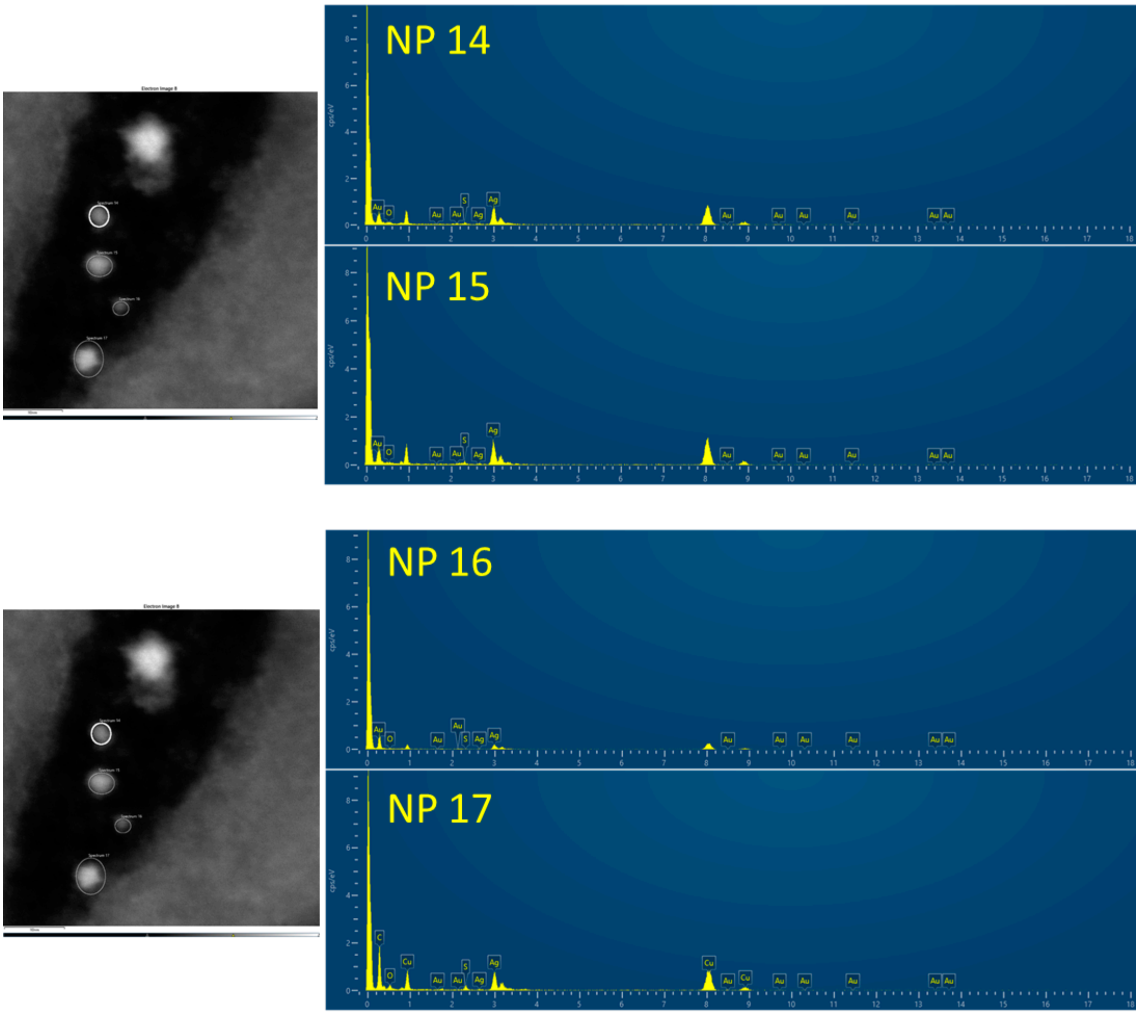

Figure S7. EDS spectra of selected Ag-enriched Au-Ag NPs of ca. 5 nm size. (NPs 11–17 in Table S1).

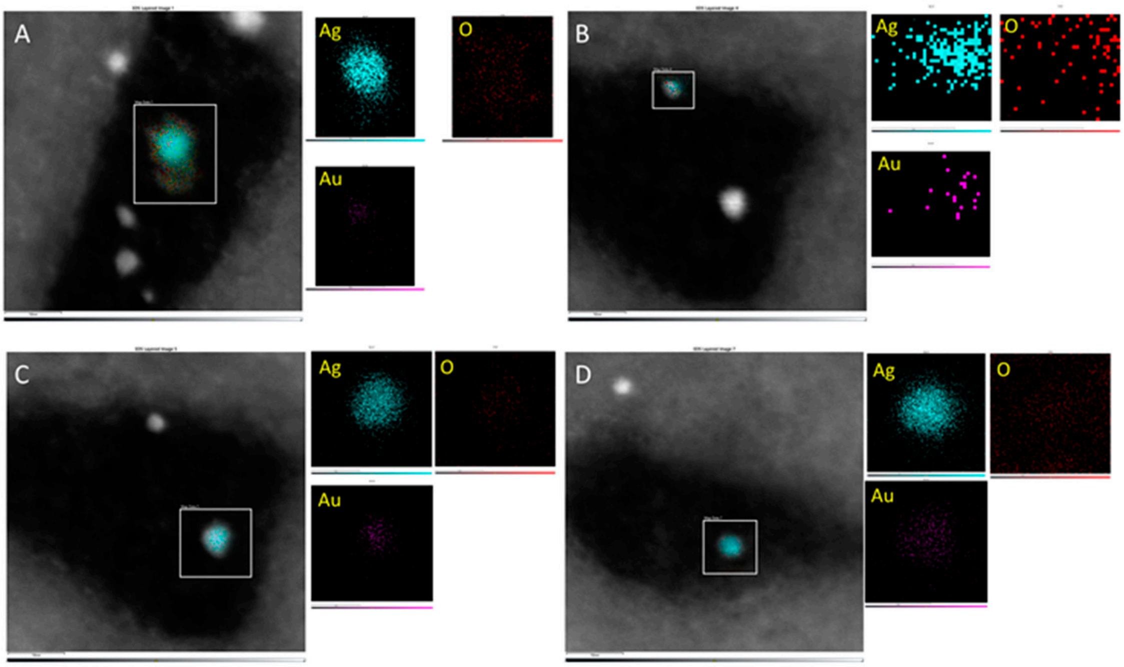

Figure S8. HAADF-STEM images of AuAg-g-C_3N_4 nanohybrid **3** (A–D) displaying Ag-enriched AuAg nanoparticles of ca. 5 nm size grafted on the surface of g-C_3N_4 and EDS individual elemental mappings for elements Au, Ag and O. Note that the HAADF-STEM images A-D display all elements mapping at a time. The analysis of the oxygen content discards the presence of Ag_2O species (bars correspond to 10 nm).

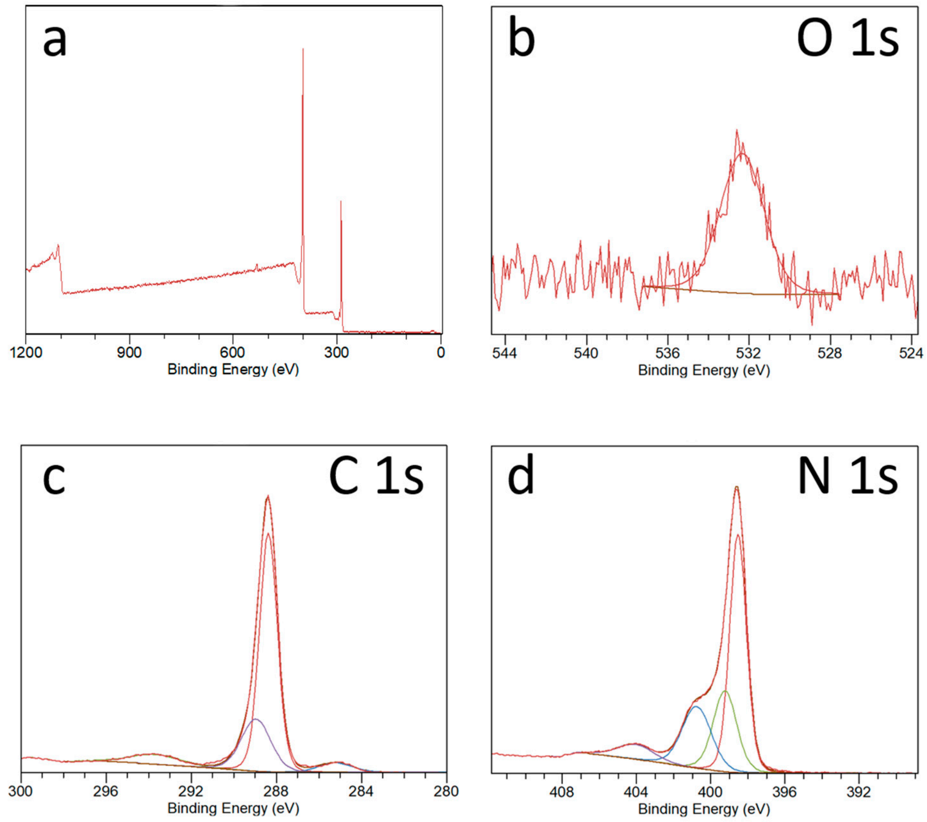

Figure S9. Wide XPS spectrum for pristine g-C_3N_4 (a). High-resolution XPS spectra for O 1s (b) C 1s (c), N 1s (d).

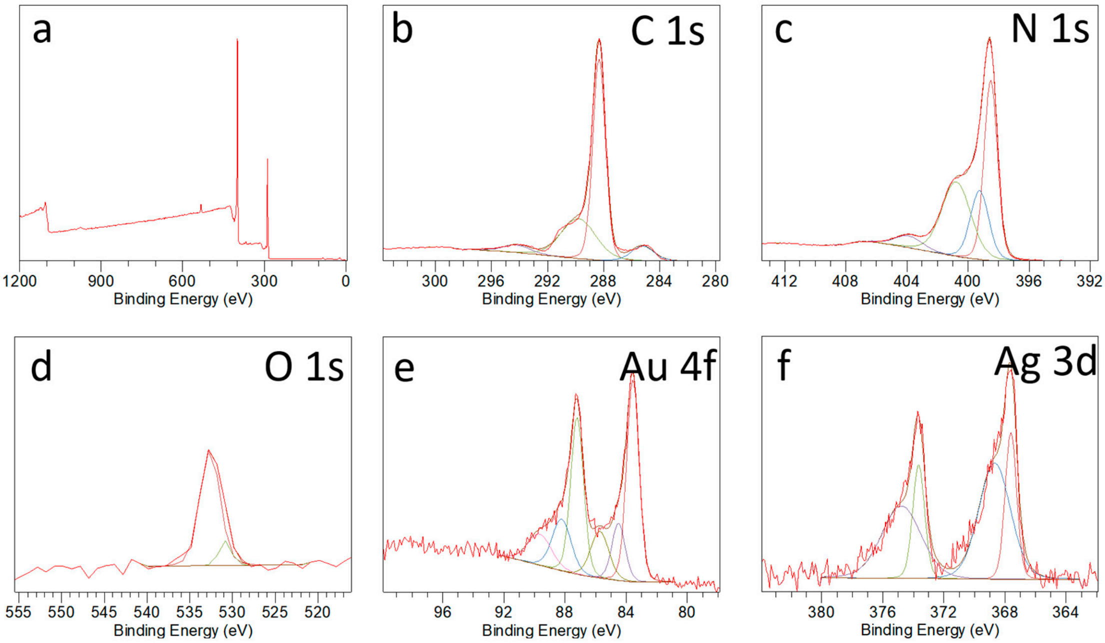

Figure S10. Wide XPS spectrum for AuAg- g-C_3N_4 nanohybrid **2** (**a**). High-resolution XPS spectra for C 1s (**b**) N 1s (**c**), O 1s (d), Au 4f (**e**), Ag 3d (**f**).

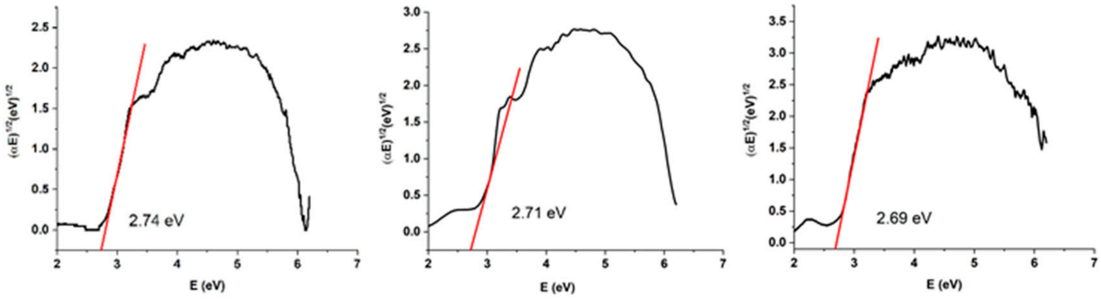

Figure S11. Tauc plots for **1–3.**

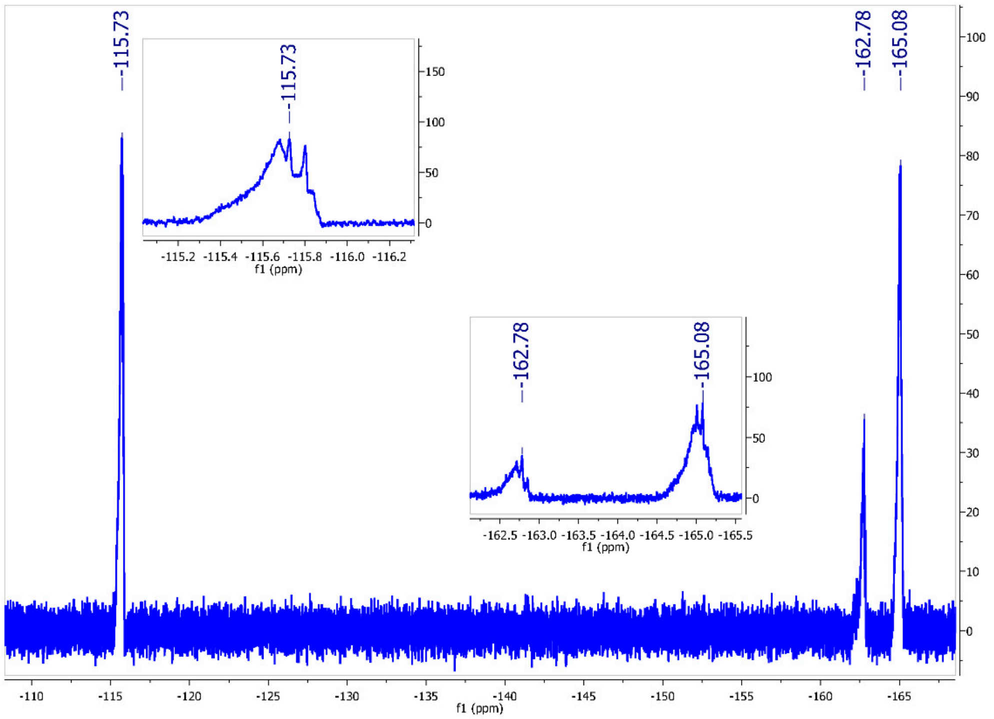

Figure S12. 19F NMR spectrum of the in situ formation of AuAg-g-C_3N_4 nanohybrid **3**. The obtained profile is assigned to the presence in solution of the molecule C_6F_5-C_6F_5 as a byproduct of the reduction of Au(I) and Ag(I) ions to the zeroth oxidation state..

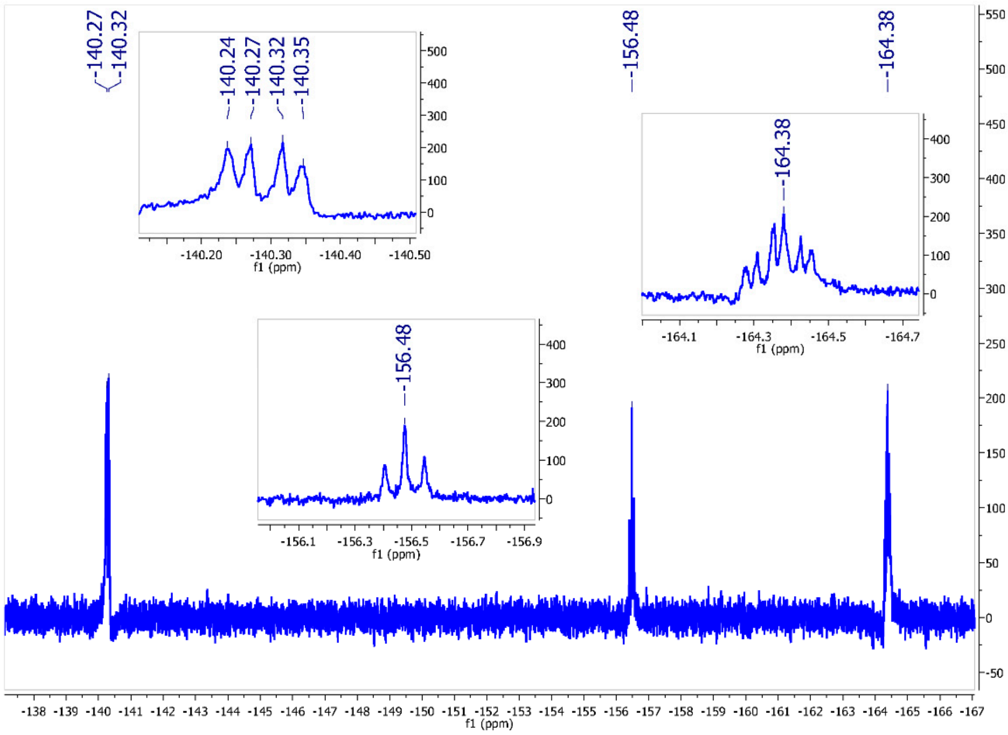

Figure S13. ^{19}F NMR spectrum of the reaction mixture of complex $[Au_2Ag_2(C_6F_5)_4(OEt_2)_2]_n$ and g-C3N4. The presence of broad signals assigned to $[Au(C_6F_5)_2]^-$ units in solution agrees with the dissociation of the precursor into its ionic components $[Au(C_6F_5)_2]^-$ and $[Ag(OEt_2)]^+$, as expected when a donor solvent such as ethyleneglycol is used.

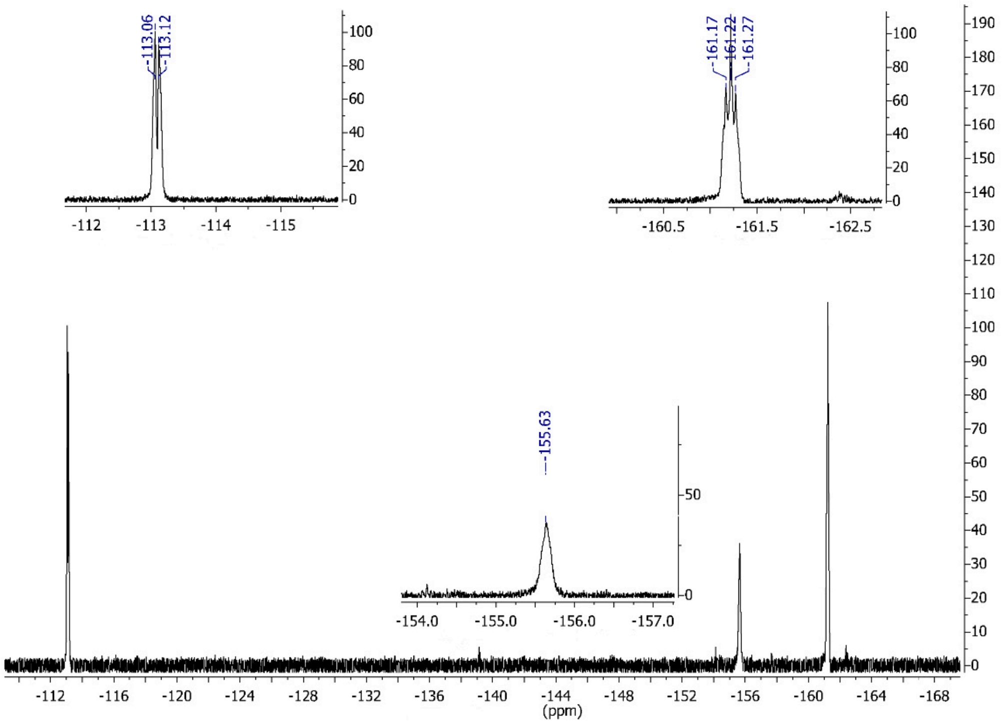

Figure S14. ^{19}F NMR spectrum of the in situ reaction between complex $[Au_2Ag_2(C_6F_5)_4(OEt_2)_2]_n$ in the presence of melamine in a 4:2 metals:melamine ratio. The obtained profile is assigned to the presence of $[Au(C_6F_5)_2]^-$ units in solution, in agreement with the coordination of silver to melamine.

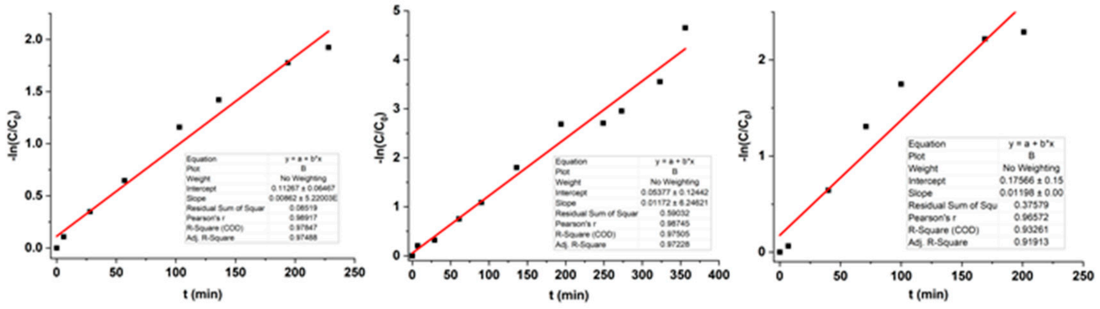

Figure S15. Kinetics of the photocatalysts 1 (left), 2 (center) and 3 (right) under visible light. The fitting results are represented assuming a pseudo-first order reaction.

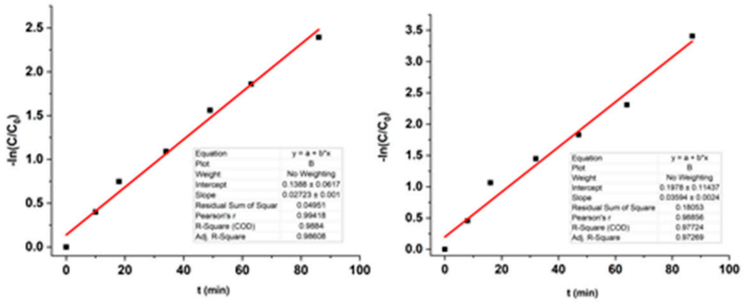

Figure S16. Kinetics of the photocatalysts **1** (left), and **3** (right) under sunlight. The fitting results are represented assuming a pseudo-first order reaction.
